# Unusual Cases

**Published:** 1850-06

**Authors:** Frank H. Hamilton

**Affiliations:** Professor of Surgery in the University of Buffalo, and one of the Surgeons to the Buffalo Hospital of the Sisters of Charity


ART. II—*Unusual Cases, reported by* FRANK H. HAMILTON, *Professor of Surgery in the University of Buffalo, and one of the Surgeons to the Buffalo Hospital of the Sisters of Charity.*

*Maggots in the Ear.*—July 14, 1849. Jonah Harvey, aged 24 years, a [illegible]rick-maker, called upon me and gave the following account of himself. On [illegible]e 12 inst., at 2 P. M., a "common fly" got into his ear, and in his at[illegible]mpts to dislodge it, the fly was driven farther in, where it remained about [illegible]e minutes before it was entirely removed. Within 12 hours from this time, Harvey felt something alive and moving in his ear. On the 13th, 26 hours after the fly was in his ear, several maggots dropped out, each about two lines in length. On the 14th, Dr. Lockwood, of this city, removed two, and at 12 A. M., of the same day, forty-six hours after the occurrence, I removed twenty-one, each one about five lines in length, and very active, so that probably not less than forty maggots were hatched. No more were seen after this. During the time they occupied the meatus, they occasioned constant uneasiness, and sometimes severe pain. Harvey has had a discharge from his ear for many years, which was still continuing at the time of the occurrence, and its odor probably attracted the fly, and induced it to deposit its larves. When the maggots were removed and carefully deposited in a dry box, they ceased to grow, and died in about 8 or 10 hours.

*Curious Affection of the Raphe.*—A child, aged 3 years, has phymosis and adhesion of the prepuce to the glans penis. There exists, also along the raphe of the scrotum, extending from an inch in front of the anus to the glans penis, an elevated sinus with transparent walls of about the size of a crow quill, closed at both ends, and nearly filled with a whitish, cream-like fluid, which can be seen to pass from one part to another when pressure is made. I opened the sinus at one point, and expressed some of the fluid. The parents had discovered it some time since, but cannot say whether it was congenital.

*Deaf but not Dumb.*—Henry Streeter, aged 3 years, Oak street, Buffalo, an intelligent looking child. Until three weeks since he could talk "well and plain." He then had a fever, accompanied with convulsions. He has had a discharge from both his ears since he was one year old, which still continues, and was unabated during the fever. He complained, much of the time during the fever, of his head.

Two weeks have now elapsed since his complete recovery, and he has not spoken a word. He answers promptly and correctly, all questions put to him, even when addressed in a somewhat low tone, but his answers are made only by intelligent signs. He laughs and shouts, but does not articulate sounds.

In the second vol., p. 574 of this Journal, I have reported the case of a child, aged eight years, who was intelligent and healthy, and whose hearing was perfect; she could utter simple sounds, but was unable to articulate more than eight or ten words.

*Hair out of Place.*—John Armstrong, corner of Eagle and Cedar streets, Buffalo, aged 38, a stucco worker. First noticed, about two weeks before he called on me, an itching on his foot ; on examination, he found a black hair of about the size and color of a cat's "feeler," projecting from the top of the foot, opposite the tarso-phalangeal articulation of the little toe. The thickest part of the hair was outward, and it was about an inch and a quarter in length. He seized it, and attempted to pull it out. He pulled quite hard, until it lifted up the skin and gave him considerable pain, when it broke off about a quarter of an inch above the skin. After this, it began to pain him, and now (spring of 1848,) the whole foot is swollen, red and œdematous, and especially tender about the joint of the little toe. It hurts him much to move the joint."

April 1850, I saw him again, and he says the remainder of the hair worked its way through on the bottom of the foot about six weeks after he called on me ; the escape of the hair being preceded by a small pustule. It was about an inch and a quarter in length, and sharpened at the end which first appeared below. The joint, and parts in its neighborhood are still tender.

Armstrong says a cat with black "feelers," he has occasionally found at his feet in bed, and this hair resembled in color, size, &c., the "feelers" of this cat. He believes it was one of them. He is a man in good circumces, and was wearing at the time, woolen socks and high boots. He is an Englishman by birth, and left England 16 years ago and took up his residence in Georgia, and remained either there or in South Carolina about four years ; has seen, and often cut out from others the "filiaria," and knows this was not of that character. It had no appearance of a head and it was too firm. Besides it is now 12 years since he left the South, and he has never before had anything of the kind.

*Horn on the Face.*—Arnold Slocum, aged 50 years, keeper of "Huff's Hotel," in Buffalo; complexion florid; a good liver. His mother was fleshy and florid, and had for twenty years, or more, a brownish, scaly spot on her face, which her friends suspected was cancerous, yet it never became a sore. She died a'et 74. His Father, whom Mr. A. Slocum resembles more than his mother, died at 65, and had for many years a sore on the inside of his lower lip. About 16 years ago, the son, the subject of this notice, discovered a yellowish, branny spot on the side of his nose. It disappeared about 10 years since, but lately it has reappeared. There is also a similar spot at the outer angle of the eye, which came recently. Nine years ago, a spot precisely like this appeared about half an inch below the centre of the right, lower lid, and it remained without material change until a year since, when he pulled the crust off, and the surface bled. From this time a horn commenced growing, which has attained now 7 lines in length, narrow at its summit, and about 5 lines in breadth at its base. It has the color and firmness of horn. Around the base, the skin was elevated to about one line in breadth and height, and was vascular and sensitive.

To-day, I excised the horn freely by two elliptical incisions. It did not extend beneath the skin. The hæmorrhage was trivial.

